# Troxipide nanosuspensions prevent indomethacin‑induced gastrointestinal lesions in adjuvant-induced arthritis rats

**DOI:** 10.1186/s40780-026-00542-w

**Published:** 2026-01-24

**Authors:** Kazuya Fujimoto, Hiroko Otake, Rie Tanaka, Fumihiko Ogata, Chika Fujii, Naoki Yamamoto, Naohito Kawasaki, Toshihiko Ishizaka, Takeshi Kotake, Noriaki Nagai

**Affiliations:** 1 Faculty of Pharmacy, Kindai University, 3-4-1 Kowakae, Higashi-Osaka, Osaka 577-8502 Japan; 2 Department of Pharmacy, Sakai City Medical Center, 1-1-1, Ebaraji-cho, Nishi-ku, Sakai, Osaka 593-8304 Japan; 3 Research Promotion Headquarters, Fujita Health University, 1-98 Degakugakubo, Kutsukake-cho, Toyoake, Aichi 470‑1192 Japan

**Keywords:** Troxipide, Nanosuspension, Gastrointestinal injury, Indomethacin, Rheumatoid arthritis

## Abstract

**Background:**

Non-steroidal anti-inflammatory drugs (NSAIDs) are widely used for managing inflammatory disorders such as rheumatoid arthritis (RA); however, their long-term use is frequently associated with gastrointestinal (GI) complications, often leading to RA treatment discontinuation. To address this issue, we developed an oral formulation of troxipide (TRO), a pharmaceutical agent with gastroprotective properties, using nanosuspension technology to enhance its GI absorption and therapeutic efficacy.

**Methods:**

The TRO nanosuspensions (TRO-NP@dis) were prepared by bead milling with methylcellulose (MC) as a stabilizing agent, yielding particles with an average diameter of 148 nm. An adjuvant-induced arthritis (AA) rat model, characterized by heightened susceptibility to NSAID-induced GI injury, was employed to assess pharmacokinetics and therapeutic efficacy.

**Results:**

TRO-NP@dis showed improved dispersibility relative to conventional microparticle dispersions (TRO-MP@dis). Although the viscosities and zeta potentials of TRO-MP@dis and TRO-NP@dis were comparable, the solubility of TRO-NP@dis was markedly higher. Pharmacokinetic studies in rats revealed enhanced retention of TRO in the stomach, jejunum, and ileum following oral administration of TRO-NP@dis compared with TRO-MP@dis. Indomethacin (IND) administration (40 mg/kg) significantly increased the mucosal lesion area in AA rats compared to that in controls. However, the co-administration of TRO-NP@dis significantly reduced the lesion areas in the stomach, jejunum, and ileum compared to those in vehicle-treated AA rats, indicating notable protective effects. These findings suggested that TRO-NP@dis confers enhanced mucosal adhesion and retention, leading to improved therapeutic outcomes in patients with IND-induced GI injury.

**Conclusions:**

This nanosuspension-based delivery system represents a promising approach for mitigating NSAID-related GI complications in patients with RA by increasing local drug concentrations and reducing mucosal damage.

## Background

Among the different drug delivery routes, oral administration continues to be the most preferred approach, primarily because of its ease of use and practicality. This route is particularly advantageous in promoting patient adherence, especially in the context of long-term therapy, as it allows for self-dosing, is noninvasive, and supports flexible scheduling of medication intake. Nonsteroidal anti-inflammatory drugs (NSAIDs) are routinely administered orally for the clinical management of rheumatoid arthritis (RA) because of their effectiveness in controlling inflammation and alleviating pain. However, this route of administration is associated with adverse gastrointestinal (GI) effects that can lead to treatment discontinuation. Several studies have reported that NSAID-induced GI complications occur more frequently in patients with RA than in healthy individuals [1, 2]. To mitigate these risks, pharmaceutical agents with known gastroprotective properties have been used as adjunctive therapies. A representative example is rebamipide, which exerts its protective effects on the gastric mucosa through mechanisms such as the enhancement of endogenous prostaglandin production and increased secretion of gastric mucus, thereby promoting the healing of damaged mucosal tissue [3]. Another agent, troxipide (TRO), has demonstrated therapeutic potential for GI lesions, including gastric ulcers, and both acute and chronically exacerbated gastritis [4–7]. After oral administration of TRO, the drug reaches both the stomach and small intestine and thus exerts therapeutic effects locally at these sites. In addition, the drug absorbed from the gastrointestinal tract enters the systemic circulation and is subsequently distributed to tissues, including the stomach and small intestine, thereby contributing to its therapeutic effects. Thus, TRO exerts its effects through both local action in the gastrointestinal tract and systemic action after absorption. Its action mechanisms involve improvement of circulatory abnormalities via increased gastric mucosal blood flow, normalization of gastric mucosal components, and upregulation of prostaglandins with cytoprotective activity in the gastric mucosa [4–7]. Despite the use of these agents, the prevention and treatment of NSAID-induced GI injury remains suboptimal, and there is an urgent need to develop more effective therapeutic strategies.

The therapeutic performance of agents, such as TRO, is intricately linked to their oral absorption, which is predominantly determined by the physicochemical characteristics of the drug and the physiological conditions within the GI tract [8, 9]. The key determinants of oral bioavailability (BA) include membrane permeability, presystemic (first-pass) metabolism, dissolution kinetics, aqueous solubility, and vulnerability to efflux transporters. Unfavorable properties in these domains can significantly hinder drug transit across the GI barrier [10]. Consequently, the gastrointestinal environment involves a combination of physical, chemical, enzymatic, and biological membrane obstacles, limiting the absorption and therapeutic efficacy of poorly bioavailable compounds [11, 12]. In light of these challenges, one of the most promising strategies for improving drug absorption involves the application of nanoscale delivery systems to enhance solubility, chemical stability, and transmembrane transport. These nanosystems offer an advanced platform for delivering compounds with low solubility and limited permeability by facilitating their passage through complex gastrointestinal barriers [13].

Nanomedicine has garnered considerable interest owing to the multifunctional capabilities of nanoparticles (NPs), including the protection of therapeutic agents from external degradation, targeted delivery to specific tissues, and sustained drug release profiles [14]. However, to achieve effective oral delivery, nanomedicines must traverse a series of physicochemical and biological barriers present in the GI tract, such as the acidic gastric environment, pH fluctuations, and enzymatic degradation by proteases throughout the GI lumen [15, 16]. Varying the pH along the GI tract can significantly influence their ionization states, structural morphology, and ultimately the functional performance of NPs containing ionizable functional groups (e.g., weak acids or bases) [17]. When targeting specialized intestinal immune structures such as Peyer’s patches or accessing the lamina propria, the intestinal epithelial barrier and its overlying mucus layer serve as significant obstacles to NP penetration [11, 18]. Therefore, a rational NP design may involve engineering surface properties that promote mucoadhesion and enable transport through the epithelial lining. Conversely, for NPs intended to release drugs into the small intestine, which is the primary site of nutrient and drug absorption, the relatively short transit time (approximately 3–4 h) imposes temporal constraints on drug release and absorption, posing additional formulation challenges [19]. Drug solubility is strongly influenced by particle size; larger particles possess lower surface-area-to-volume ratios, resulting in reduced interactions with the solvent and slower dissolution rates. A well-established approach for enhancing dissolution involves reducing the particle size, thereby increasing the available surface area. Several nanosuspension-based oral formulations have already reached the market, offering improved dissolution and absorption characteristics [20]. Nanosuspensions are typically defined as colloidal dispersions composed of submicron-sized drug particles stabilized by surfactants and are generally prepared using techniques such as wet milling or high-pressure homogenization. We previously established a method for the preparation of drug NPs with particle sizes below 100 nm by combining cellulose-based compounds using a wet-bead milling technique [21, 22]. Furthermore, we reported that this nanosuspension approach led to an increase in drug solubility, as predicted by the Ostwald–Freundlich equation [23]. In addition, we demonstrated that this nanonization technology enhanced the GI residence time and absorption of drugs [24], thereby contributing to improved pharmacological efficacy. Moreover, NPs have been reported to demonstrate increased affinity for tissue surfaces, potentially leading to greater tissue retention and absorption than would be expected based solely on their solubility [22, 24].

In this study, we applied this nanocrystallization technique to TRO to investigate its effect on GI retention. Moreover, we evaluated the therapeutic potential of the resulting formulation in a GI injury model induced by the excessive administration of indomethacin (IND), a representative NSAID, in an adjuvant-induced arthritis (AA) rat model that mimicked the pathological features of RA.

## Methods

### Animals

Seven-week-old male Dark Agouti (DA) rats, weighing 152 ± 5.1 g, were obtained from Shimizu Laboratory Supplies Co., Ltd. (Kyoto, Japan). The rats were maintained under controlled environmental conditions (temperature: 25 °C; light/dark cycle: 12 h; lights on from 07:00 to 19:00). Standard laboratory chow (CE-2; Clea Japan Inc., Tokyo, Japan) and water were provided ad libitum throughout the study. To induce arthritis, a suspension consisting of 10 mg/mL heat-inactivated *Mycobacterium butyricum* (Difco; Detroit, MI, USA) was added to Bayol F oil. Next, 50 µL of this adjuvant suspension was administered subcutaneously into the plantar surface of the right hind paw and the base of the tail of DA rats. Control animals (non-arthritic group) received 50 µL of Bayol F oil alone. AA progression was evaluated by quantifying paw inflammation. Paw edema, a key indicator of the inflammatory response, was measured using a plethysmometer. The degree of swelling was calculated using the following formula (Eq. 1):

1 $$\begin{aligned}\mathrm{Paw}\:\mathrm{edema}\left(\Delta\mathrm{mL}\right)&=\mathrm{Paw}\:\mathrm{volume}\:\mathrm{of}\:\mathrm{arthritis}\:\mathrm{rat}\cr&\quad-\mathrm{paw}\:\mathrm{volume}\:\mathrm{of}\:\mathrm{normal}\:\mathrm{rat}\end{aligned}$$

All experimental procedures involving animals were performed in strict accordance with the institutional guidelines established by Kindai University, standards set by the Japanese Pharmacological Society, Guide for the Care and Use of Laboratory Animals published by the National Institutes of Health, Animal Research: Reporting of In Vivo Experiments (ARRIVE) guidelines, and 2020 edition of the American Veterinary Medical Association (AVMA) Guidelines for the Euthanasia of Animals. The experimental protocol was approved by the Animal Experimentation Committee of Kindai University (approval no. KAPS-2022-010; date of approval: April 1, 2022). Throughout the study, the general health status and behavioral responses of the animals were monitored daily. An adjuvant was administered under anesthesia to minimize pain and distress. Euthanasia was conducted in accordance with the experimental timeline, as none of the rats reached the predefined humane endpoints, necessitating early termination. Euthanasia was performed by injecting pentobarbital (200 mg/kg, i.p.) according to the AVMA guidelines 2020.

### Chemicals

TRO, IND, methanol, and Bayol F oil were purchased from Wako Pure Chemical Industries Ltd. (Osaka, Japan). Methylcellulose SM-4 (MC) was provided by Shin-Etsu Chemical Co., Ltd. (Tokyo, Japan). Heat-killed *M. butyricum* was obtained from Difco (Detroit). A protein assay kit and pentobarbital were obtained from Bio-Rad (Hercules, CA, USA) and Tokyo Chemical Industry Co. Ltd. (Tokyo, Japan), respectively. All the chemicals used were of the highest purity.

### Preparation of TRO dispersions

TRO (75 mg) and MC (75 mg) were co-milled for 30 min using an agate mortar and pestle. Subsequently, 100 mg of the milled mixture was suspended in purified water to obtain a 0.5% (w/v) TRO suspension. After dispersion using a vortex mixer, the suspension was transferred into a 2 mL microcentrifuge tube containing 2 g of zirconia beads (diameter: 0.1 mm) and subjected to bead milling using a Shake Master NEO (Bio-Medical Science Co., Ltd., Tokyo, Japan) at 1,500 rpm for 3 h at 4 °C. The resulting suspension was centrifuged at 800 *g* for 60 s at 4 °C (TOMY, Japan). The prepared dispersion was referred to as TRO-NP@dis. For comparison, a suspension of raw TRO powder in a vehicle containing 0.5% MC and purified water was prepared and denoted as TRO-MP@dis.

### Measurement of TRO levels

The TRO dispersions were dissolved and used to analyze the TRO concentration. The biological samples were homogenized in methanol and centrifuged at 20.4 × 10^3^ *g* and 4 °C for 20 min. The supernatants were used as samples to measure the TRO content. A 50 µL aliquot of the sample and 100 µL of internal standard (methyl parahydroxybenzoate) were transferred into a sample tube and analyzed using high-performance liquid chromatography (HPLC). The mobile phase consisted of potassium dihydrogen phosphate and acetonitrile at a 9:1 (v/v) ratio. Chromatographic separation was performed using an LC-20AT HPLC system (Shimadzu Corp., Japan), equipped with a CTO-20AC column oven maintained at 35 °C, and an Inertsil ODS-3 column (2.1 × 50 mm; GL Sciences Inc., Japan). The flow rate of the mobile phase was set to 0.25 mL/min. Detection was conducted at a wavelength of 254 nm with an injection volume of 10 µL, using an SIL-20AC auto-injector. The total run time was 18 min. The TRO chromatographic peak was observed approximately 4 min after injection. A calibration curve (y = 0.1502x–0.0077) was generated (R² = 0.9983). The limit of detection of TRO was 0.05 µg/mL.

### Particle size distribution of TRO dispersions

The particle size distributions of TRO-MP@dis and TRO-NP@dis were measured using SALD-7100 (Shimadzu Corp., Kyoto, Japan) and Nanoparticle Tracking Analysis NanoSight LM10 (Quantum Design Japan; Tokyo, Japan), respectively. The refractive index was set at 1.60–0.10, and the particle size distribution of TRO-MP@dis was measured by SALD-7100. To measure the particle size distribution of TRO-NP@dis, the wavelength, viscosity, and time for measurement were 405 nm, 1.45 mPa∙s, and 60 s, respectively. Scanning probe microscopy (SPM) images of TRO-NPs in the dispersions, analyzed from the SPM phase-to-height images, were obtained using an SPM-9700 (Shimadzu Corp., Kyoto, Japan).

### Drug solubility and viscosity of TRO dispersions

The TRO dispersions were separated into soluble and non-solubilized forms by centrifugation at 1 × 10^5^ *g* using a Beckman Optima™ MAX-XP Ultracentrifuge (Beckman Coulter; Osaka, Japan). The TRO content in the dissolved samples was measured using HPLC as described above. The viscosity of TRO dispersions was measured using an SV-1 A viscometer (A & D Company Limited; Tokyo, Japan) at 22 °C for 3.5 min.

### Crystalline form of TRO dispersions

The crystalline forms of TRO in the TRO-MP@dis and TRO-NP@dis were measured using an XRD analyzer Mini Flex II (Rigaku Co., Tokyo, Japan) at a scanning rate, 10°/min; diffraction angles of 5°–60°; X-rays at 30 kV and 15 mA. Dried samples were obtained by volatilizing the dispersion solvent using a TAITEC VC-15SP CENTRIFUGAL CONCENTRATOR (Aich, Japan).

### TRO content in the stomach and intestine of rats

TRO@dis was orally administered to rats that had been fasted for 8 h at a dose of 2 mg/kg. At predetermined time points (3, 6, and 24 h post administration), the rats were euthanized using a lethal dose of pentobarbital (200 mg/kg). Mucosal tissues from the stomach, jejunum, and ileum were carefully excised and immediately processed. Each mucosal sample was homogenized in ice-cold methanol to extract TRO, and the resulting homogenates were centrifuged at 20,400 *g* for 15 min at 4 °C. Supernatants were collected for analysis. TRO concentrations were quantified using a previously described HPLC method. The total protein content in each sample was determined using a commercial Protein Assay Kit. The amount of TRO in the gastric, jejunal, and ileal mucosa was normalized to the protein content and expressed as nanograms per gram of protein.

### Measurement of IND-induced GI injury

TRO@dis was orally administered at a dose of 2 mg/kg 14 days after adjuvant injection. TRO@dis was administered 6 h after administration of an excessive dose of IND (40 mg/kg) to evaluate the therapeutic potential of TRO against IND-induced gastric damage, and the rats were euthanized using a lethal dose of pentobarbital (200 mg/kg) 18 h post-TRO administration. The formulation was administered 24 h after IND overdose (40 mg/kg) to assess the effects of TRO@dis on IND-induced injuries in the jejunum and ileum, followed by euthanasia via a lethal dose of pentobarbital (200 mg/kg) 24 h later. The stomach, jejunal, and ileal mucosal tissues were harvested and fixed in 10% formalin, and the lesion areas were documented using digital imaging. Lesion size was quantified using the ImageJ software. The extent of injury was expressed as the percentage ratio of the lesion area to the total mucosal surface area. Anatomical definitions for the segments of the small intestine were as follows: the duodenum was defined as the distal 2 cm of the stomach; the jejunum, averaging 26.8 ± 1.8 cm in length, corresponded to the proximal 40% of the small intestine excluding the duodenum; and the ileum, measuring approximately 40.5 ± 2.9 cm, comprised the distal 60% of the small intestine beyond the duodenum (*n* = 20).

### Statistical analysis

Statistical analyses were performed using the JMP software version 5.1 (SAS Institute Inc., Cary, NC, USA). An unpaired Student’s *t*-test was used to compare two independent groups. In cases involving more than two groups, a one-way repeated-measures analysis of variance (ANOVA) was conducted, followed by post-hoc multiple comparisons using the Tukey–Kramer test to identify significant group differences. Statistical significance was set at a threshold of *P* < 0.05. All results are expressed as the mean ± standard error of the mean (SEM).

## Results

### Preparation of TRO-NP@dis and its intestinal absorption

**Fig. 1:**
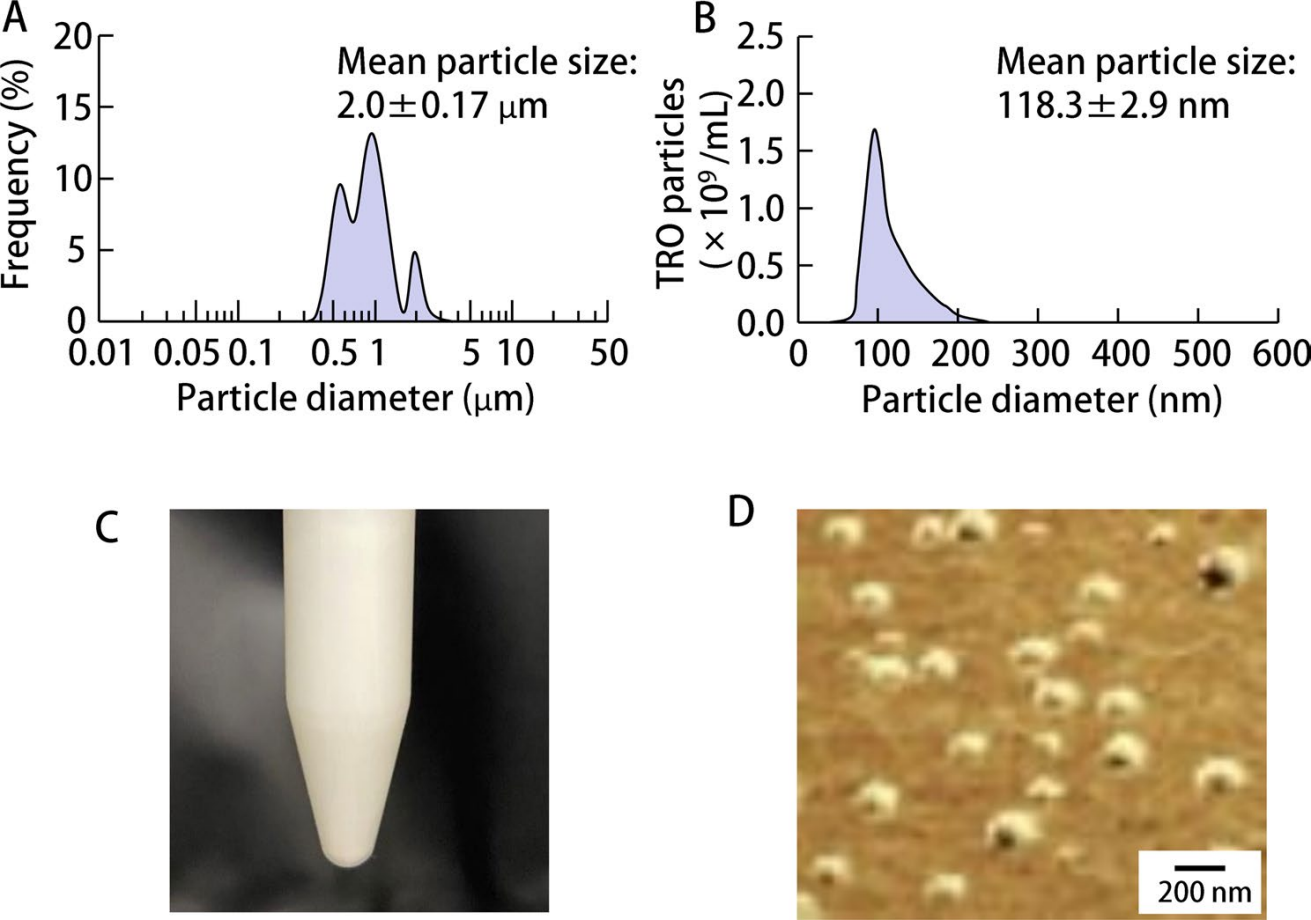
Particle size distribution of TRO with and without bead mill treatment. (**A**) and (**B**) Particle size of (**A**) TRO-MP@dis and (**B**) TRO-NP@dis. (**C,** **D**) Digital images (**C**) and SPM images (**D**) of TRO-NP@dis. The mean particle size of TRO-MP@dis and TRO-NP@dis was 2.0 μm and 118.3 nm

**Fig. 2:**
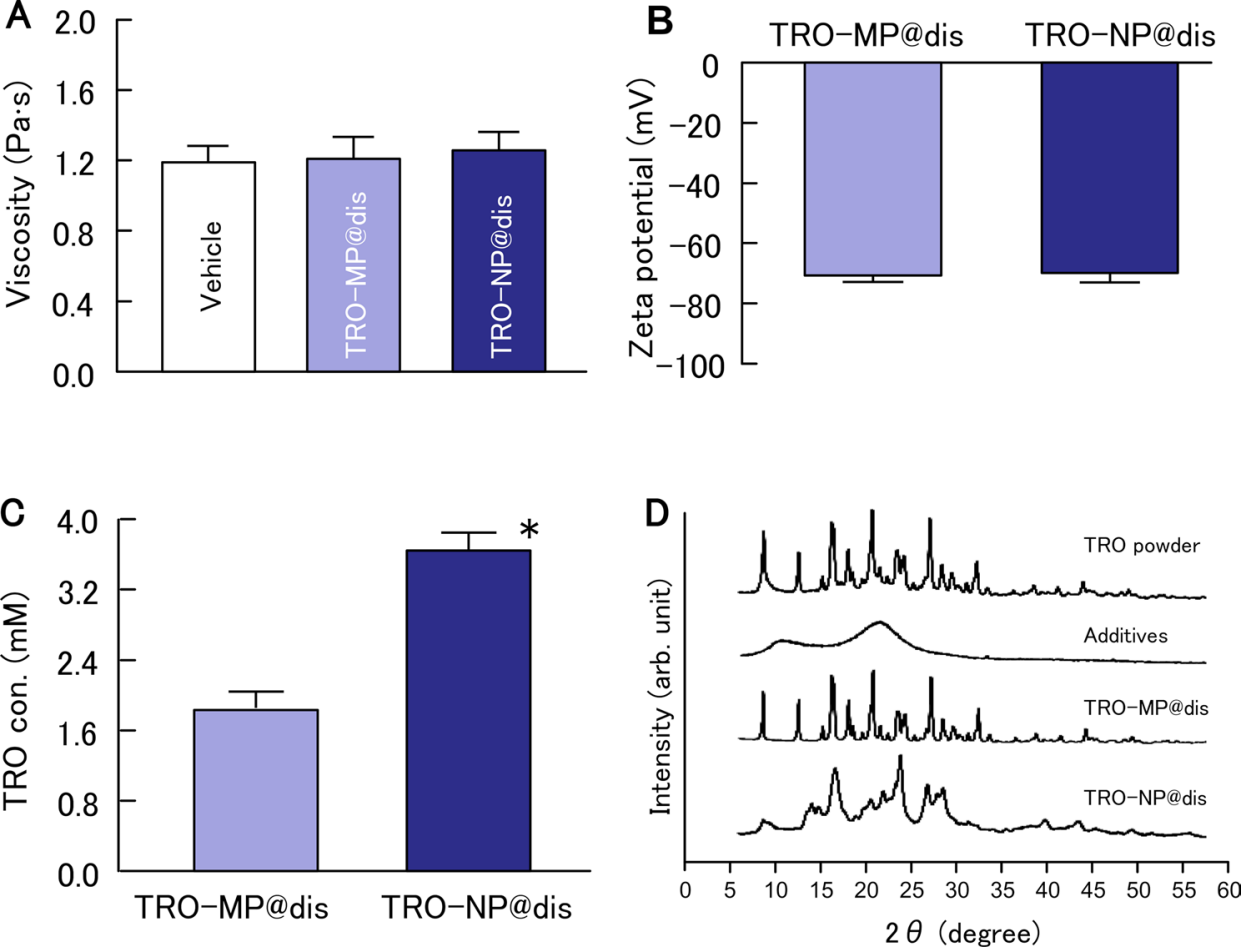
Viscosity (**A**), zeta potential (**B**), solubility (**C**), crystalline form (**D**) of TRO-MP@dis and TRO-NP@dis. The viscosities and zeta potentials of TRO-MP@dis and TRO-NP@dis were similar. In contrast, the solubility of TRO-NP@dis was higher than that of TRO-MP@dis. The crystalline form of TRO-NP@dis showed that the solid phase retains its crystalline state, thought its intensity had decreased. Means ± S.E. n = 6. *P < 0.05 vs. TRO-MP@dis for each category

**Fig. 3:**
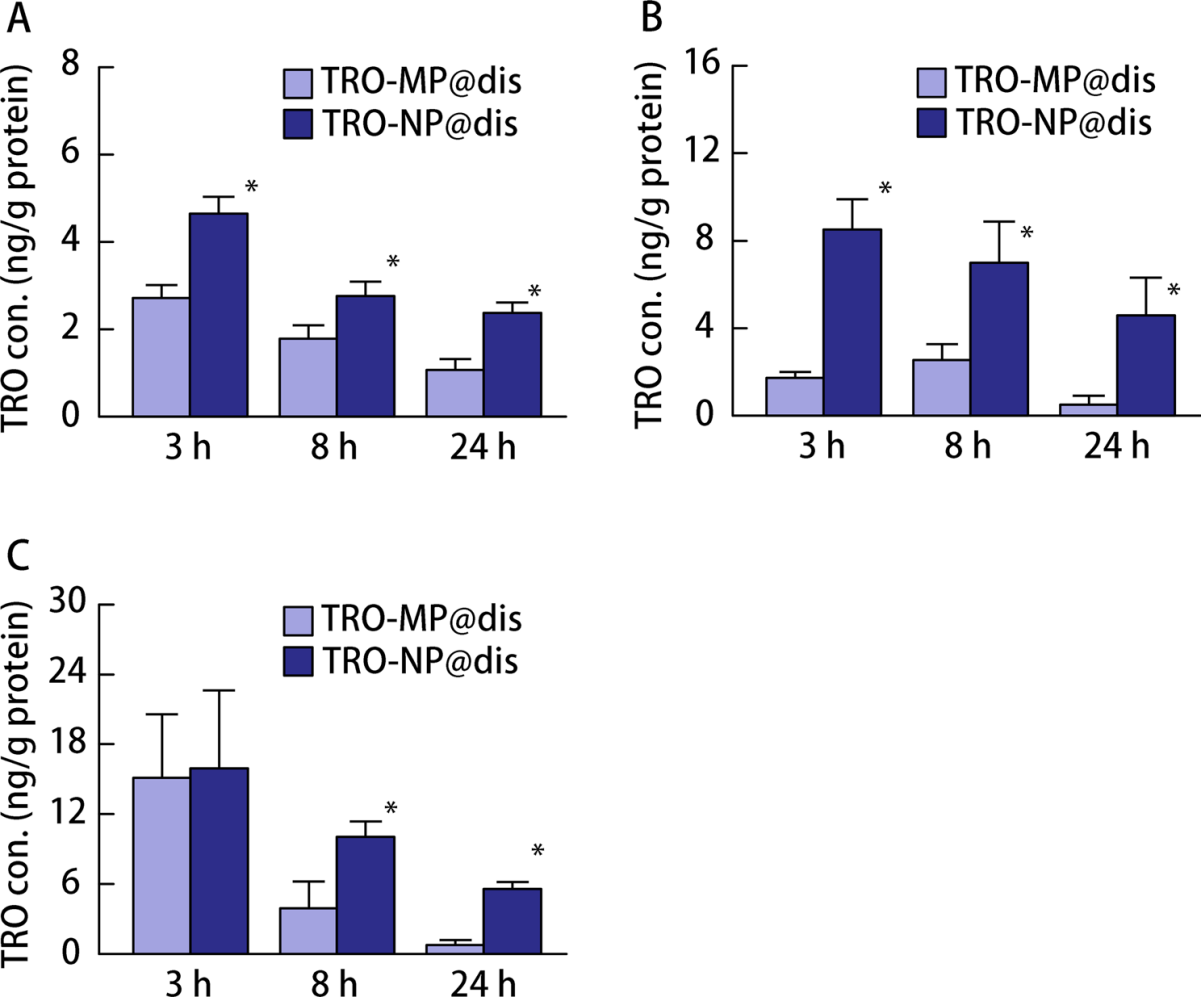
TRO content in the mucosal membrane of the stomach (**A**), jejunum (**B**), and ileum (**C**) of AA rats treated with TRO-MP@dis and TRO-NP@dis. Rats were fasted for 6 h before the experiments; however, they had free access to water. Open columns: TRO-MP@dis; closed columns: TRO-NP@dis. Means ± S.E. *n* = 6. ^*^P < 0.05 vs. TRO-MP@dis for each category. The TRO content in the stomach, jejunum, and ileum of rats treated with TRO-NP@dis was higher than that of TRO-MP@dis

First, the formulation of TRO-NP@dis was attempted. Figure 1 shows the particle size distributions of TRO with and without bead milling. The mean particle size of TRO without bead milling (TRO-MP@dis) was approximately 2 μm (Fig. 1A), whereas bead milling significantly reduced the size to 118.3 nm (Fig. 1B). Furthermore, digital imaging confirmed a well-dispersed state, and SPM images revealed spherical NP consistent with the particle size measurements (Fig. 1D). We evaluated the viscosity, zeta potential, solubility, dispersibility, and crystalline form of the formulations. The viscosity and zeta potential of TRO-MP@dis and TRO-NP@dis were comparable, approximately 1.2 Pa·s and − 70 mV, respectively (Fig. 2A and B). The solubility was significantly enhanced in the NP formulation; TRO-NP@dis was determined to be 3.6 mM (Fig. 2C). In contrast, a 0.5% (17 mM) TRO formulation was used in this study, approximately 79% of the drug existed in the solid state within the suspension. Moreover, X-ray diffraction (XRD) data of TRO-NP@dis showed that the solid phase retains its crystalline state, thought its intensity had decreased (Fig. 2D). Figure 3 illustrates the tissue concentrations of TRO in the GI tract of rats from 3 to 24 h after oral administration of either TRO-MP@dis or TRO-NP@dis. TRO was detected in the GI tissue, and its concentration was significantly higher in rats administered with TRO-NP@dis than in those administered with TRO-MP@dis. Peak concentration was observed 3 h after administration. At this time point, the TRO concentrations in the stomach, jejunum, and ileum of rats administered TRO-NP@dis were 1.7-, 4.9-, and 1.1- times higher, respectively, than those observed in the TRO-MP@dis group (Fig. 3). Moreover, sustained levels of TRO were more pronounced in the TRO-NP@dis group; 24 h post-administration, the concentrations of TRO in the stomach, jejunum, and ileum were 2.2-, 8.9-, and 7.1- times higher, respectively, than those in the TRO-MP@dis group.

### Therapeutic effect of TRO-NP@dis on IND‑induced GI lesion in AA rats

**Fig. 4:**
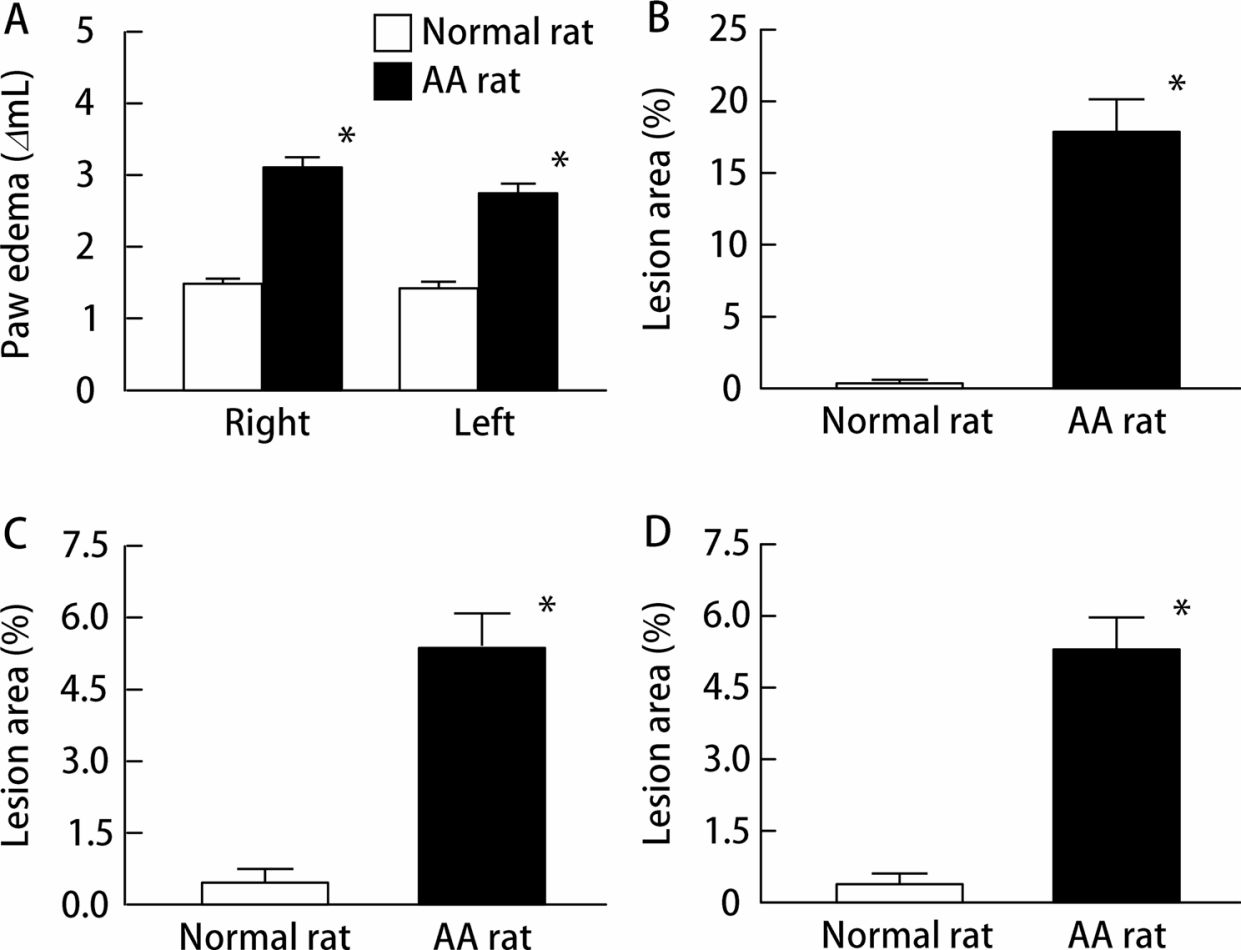
Changes in the paw edema (**A**), gastric (**B**), and ulcerogenic lesions in the jejunum (**C**) and ileum (**D**) of IND-administered AA rats. Open columns: normal rats; closed columns: AA rats. Means ± S.E. *n* = 5. ^*^P < 0.05 vs. normal rat for each category. The paw edema and lesion area in the stomach, jejunum, and ileum of IND-administered AA rats were increased in comparison with those in IND-administered normal rats

**Fig. 5:**
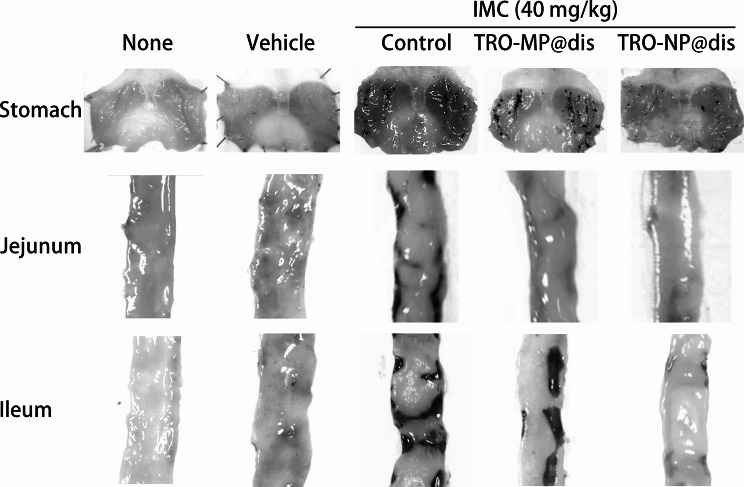
Images of gastric and ulcerogenic lesions in the jejunum and ileum of IND-treated AA rats treated with TRO-MP@dis and TRO-NP@dis. Gastric and ulcerogenic lesions were prevented by TRO-NP@dis treatment

**Fig. 6:**
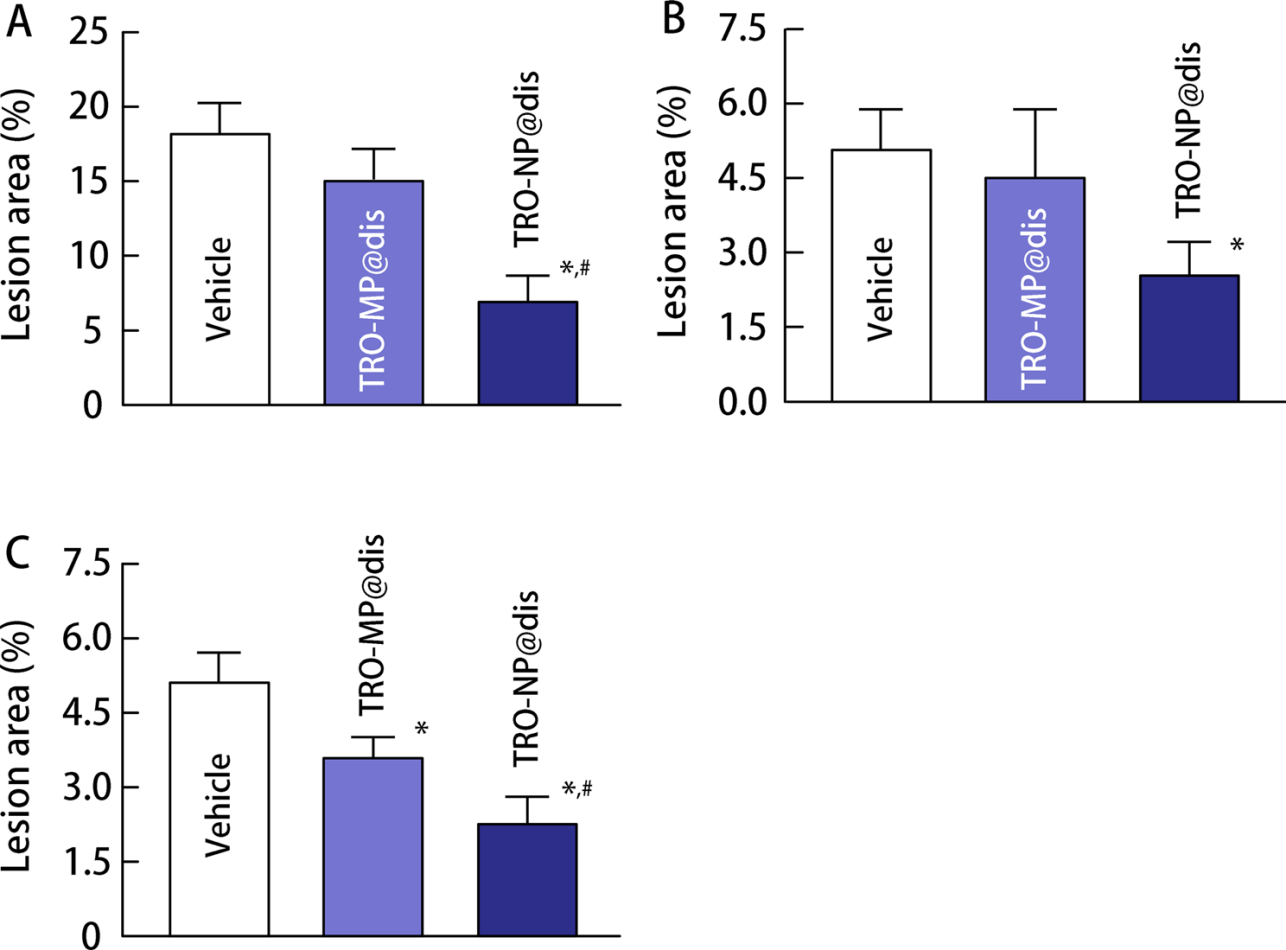
Changes in the gastric (**A**) and ulcerogenic lesions in the jejunum (**B**) and ileum (**C**) of IND-administered AA rats treated with vehicle, TRO-MP@dis, and TRO-NP@dis. Means ± S.E. *n* = 6. ^*^P < 0.05 vs. vehicle for each category. ^#^*P* < 0.05 vs. TRO-MP@dis for each category. The lesion area in the stomach, jejunum, and ileum of IND-administered AA rats treated with TRO-NP@dis significantly decreased than that of corresponding vehicle-treated AA rats

NSAID-induced GI injuries occur more frequently in patients with RA. Accordingly, a widely used animal model of NSAID-induced GI injury involves AA rats treated with a high dose of IND. We used an established model to evaluate the therapeutic effects of TRO-NP@dis on NSAID-induced GI injury. Figure 4 illustrates the induction of arthritis (Fig. 4A) and the extent of GI injury following IND overdose (Fig. 4B-D). On day 14 after adjuvant administration, paw volume, an index of arthritis severity, increased significantly in the AA group (Fig. 4A). Concurrently, the severity of GI lesions in the stomach, jejunum, and ileum was 50.9-, 11.5, and 14.1 times greater, respectively, than that observed in healthy control rats (Figs. 4B–D and 5). The therapeutic effects of TRO@dis on IND-induced GI injury are shown in Fig. 6. The administration of TRO-MP@dis partially improved ileal lesions, whereas lesions in the stomach and jejunum showed only a modest trend toward recovery. In contrast, IND-administered AA rats treated with TRO-NP@dis exhibited the most rapid and pronounced healing among all the groups. The ulcerated areas in the stomach, jejunum, and ileum of rats in the TRO-NP@dis group were reduced to 37.8%, 50.1%, and 44.2%, respectively, compared with the corresponding values in rats in the untreated group (Fig. 6).

## Discussion

**Fig. 7:**
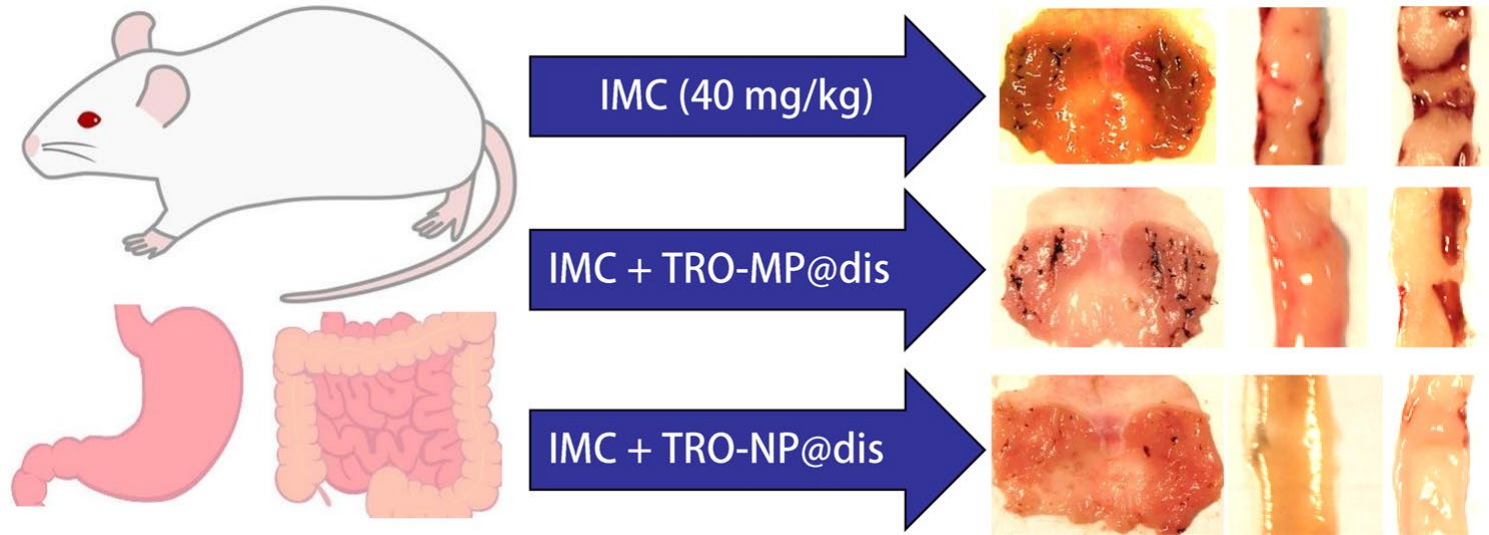
Scheme for the therapeutic effect of TRO@dis on gastric and ulcerogenic lesions of IND-administered AA rats

GI complications, which are well-known adverse effects of NSAIDs, have been reported to occur more frequently in patients with RA, often leading to treatment discontinuation. Therefore, the development of effective strategies for the prevention and treatment of NSAID-induced GI injuries is highly desired. We focused on the nanosuspension technology, which is expected to enhance drug absorption, and successfully prepared a TRO nanosuspension. Our findings demonstrated that this formulation improved both the GI residence time of the drug and its distribution to the target tissue. Moreover, the TRO nanosuspension exhibited a significant therapeutic effect against IND-induced GI injury in rats with AA, indicating its potential as a promising agent for managing NSAID-induced GI complications (Fig. 7).

Numerous studies have demonstrated that NP formulations can enhance the oral BA of poorly water-soluble, water-soluble, and biologically derived therapeutics through diverse mechanistic pathways [25–27]. Currently, a number of marketed oral pharmaceutical products based on nanosuspension platforms have been developed to facilitate improved drug dissolution and absorption characteristics [20]. Nanosuspensions are typically characterized as colloidal systems comprising submicron-sized drug particles stabilized by the incorporation of specific excipients. Nanosuspension formulation generally relies on two principal strategies: bottom-up and top-down [28]. The bottom-up approach involves constructing NPs through molecular-level assembly processes such as precipitation, melt emulsification, and microemulsion techniques. In contrast, the top-down methodology entails the mechanical size reduction of larger particles into nanoscale dimensions via processes such as high-pressure homogenization as well as comminution techniques including ball milling and jet milling [29].

These nanosuspension-based formulations confer multiple pharmaceutical benefits [30], including improved physicochemical stability, enhanced solubility, systemic BA of active compounds, potential for dose reduction, and suitability for the formulation of hydrophobic drug entities. We have previously demonstrated that NPs can be successfully produced using a bead mill in the presence of MC, which is a top-down technology [22, 31]. Based on these findings, we investigated the effect of MC-assisted bead milling on the particle size of TRO (3,4,5-trimethoxy-N-(3-piperidyl) benzamide), a gastroprotective agent with antiulcer, anti-inflammatory, and mucus-secreting properties. Here, we successfully prepared TRO-NP@dis (mean particle size: 118.3 nm) using bead milling (Fig. 1). The solubility of TRO-NP@dis was significantly improved by approximately two fold compared to TRO-MP@dis (Fig. 2C). In addition, the crystalline form of TRO-NP@dis showed that the solid phase retains its crystalline state, thought its intensity had decreased (Fig. 2D). The decreased particle size and crystallinity resulting from nanolization using bead milling is considered to improved TRO solubility.

The extent and rate of drug absorption and the overall BA are predominantly influenced by the solubility of the drug in aqueous environments, its dissolution behavior, and its permeability across the GI membrane [32]. Aqueous solubility is particularly critical following oral administration, as the drug must be present in a dissolved, water-soluble form at the site of absorption to enable effective uptake [33, 34]. Both solubility and intestinal permeability are key determinants of in vivo absorption efficiency. Several solubility enhancement strategies have been employed to address these limitations, thereby optimizing oral BA [35]. The improved physicochemical profile (solubility) of TRO-NP@dis translated into an enhanced in vivo performance. Following oral administration, significantly higher concentrations of TRO were detected in the GI tissues of rats treated with TRO-NP@dis than in those treated with TRO-MP@dis (Fig. 3). This enhancement was observed across multiple sites, including the stomach, jejunum, and ileum, and was most prominent 3 h post-administration (Fig. 3). Moreover, the TRO-NP@dis group exhibited a more sustained tissue concentration over a 24 h period, indicating prolonged retention and possibly improved mucosal adhesion (Fig. 3).

The ability of drug particles to permeate the mucus barrier is largely governed by their physicochemical characteristics, particularly the particle size, surface charge, and surface functionalization. Particles bearing neutral or slightly negative surface charges are generally favored for effective mucus penetration and to minimize electrostatic interactions with the negatively charged mucin network [36, 37]. Because the pore size of the mucus matrix typically ranges from 100 nm to 200 nm [38], NPs smaller than this threshold are particularly promising for efficient diffusion through the mucus layer. However, the viscosity and zeta potential of the nanoparticulate formulation were comparable to those of the microparticulate formulation, suggesting that nanosizing did not negatively affect the rheological behavior or surface charge of the dispersion. However, the TRO-NP@dis prepared in this study exhibited a particle size of less than 200 nm and a zeta potential of approximately − 20 mV (Fig. 2A and B), suggesting that these characteristics may facilitate penetration into the GI mucosa, thereby potentially contributing to enhanced mucosal retention. These findings suggest that nanoparticulate TRO offers superior tissue distribution, possibly owing to its increased surface area, rapid dissolution rate, and improved interaction with the mucosal lining. The enhanced retention and tissue distribution observed with TRO-NP@dis (Fig. 3) may contribute to more consistent therapeutic outcomes, particularly in GI applications where the local drug concentration is a key determinant of efficacy.

Different experimental models have been established for studying GI injuries. Among these, the administration of NSAIDs to AA rats is a widely used model, as these rats exhibit more severe GI damage than healthy rats, resembling the pathology observed in humans [1, 2, 39, 40]. In this study, IND administration induced severe GI injury in AA rats with IND administration. Therefore, this model was used to evaluate the therapeutic efficacy of the TRO-NP@dis. These results confirmed the therapeutic potential of TRO-NP@dis in this model.

The therapeutic relevance of this improved formulation was further supported by experiments using a well-established rat model of NSAID-induced GI injury with AA. Patients with RA are at higher risk of NSAID-induced GI toxicity, and the AA rat model mimics the inflammatory and ulcerogenic environmental characteristics of such conditions. Evaluated the therapeutic effect of TRO@dis in this model, the wound area in the TRO-NP@dis group was markedly smaller, suggesting rapid and more effective healing (Fig. 6). The significant therapeutic efficacy of TRO-NP@dis further corroborated the pharmacokinetic data (Fig. 3). The TRO nanosuspension is considered to exhibit enhanced physical adhesion to tissues due to its small particle size in the solid state, which may delay its transport to the distal gastrointestinal tract by peristaltic movement through adhesion to tissue microstructures such as villi. In addition, nanosuspension formulations have been reported to enhance drug uptake into target cells via endocytic mechanisms, leading to increased local drug concentrations in the gastric and gastrointestinal tissues. Furthermore, previous studies have demonstrated that nanosuspensions can improve gastrointestinal absorption (bioavailability) owing to their physicochemical properties, and the resulting elevated systemic blood concentrations further enhance drug levels in injured tissues such as the stomach and gastrointestinal tract. Collectively, these multiple factors—(1) increased tissue adhesion in the solid state, (2) enhanced local drug concentration in gastrointestinal tissues via endocytosis, and (3) increased tissue drug levels associated with improved oral bioavailability—may be involved in the increased drug concentration in the gastrointestinal tract (Fig. 3) and the enhanced local therapeutic effects observed in Figs. 5 and 6.

Histological analysis using tissue sections, along with the evaluation of changes in inflammatory cytokines, such as nitric oxide, one of the key mediators implicated in IND-induced GI injury, is essential for elucidating the therapeutic effects of the treatment. Future studies should investigate these aspects using immunohistochemical staining.

## Conclusions

We developed an oral TRO nanoformulation and evaluated its protective effects against IND-induced GI injury. TRO-NP@dis was prepared using a bead-milling method with MC as a stabilizing agent, achieving a mean particle size of 118.3 nm and superior dispersibility compared with TRO-MP@dis. An AA rat model that mimics human RA and exhibits heightened sensitivity to IND-induced GI damage was used to assess mucosal retention and therapeutic efficacy. Following oral administration, TRO-NP@dis demonstrated significantly enhanced retention in the gastric and intestinal mucosa compared to TRO-MP@dis, possibly because of increased mucosal adhesion associated with reduced particle size. Moreover, TRO-NP@dis markedly improved the healing of both IND-induced gastric and small intestinal lesions. These findings suggest that TRO-NP@dis offers improved mucosal residence time and therapeutic efficacy, making it a promising candidate for mitigating NSAID-related GI injuries.

## Data Availability

The data generated in the present study may be requested from the corresponding author.

## Abbreviations

AA: Adjuvant-induced arthritis
ANOVA: A one-way repeated-measures analysis of variance
ARRIVE: The Animal Research: Reporting of In Vivo Experiments guideline
AVMA: The American Veterinary Medical Association Guidelines
BA: Bioavailability
DA: Dark Agouti
GI: Gastrointestinal
HPLC: High-performance liquid chromatography
IND: Indomethacin
MC: Methylcellulose
MP: Microparticle
NP: Nanoparticle
NSAIDs: Non-steroidal anti-inflammatory drugs
RA: Rheumatoid arthritis
SEM: Standard error of the mean
SPM: Scanning probe microscope
TRO: Troxipide
XRD: X-ray diffraction
